# Role of Galectin-3 in Obesity and Impaired Glucose Homeostasis

**DOI:** 10.1155/2016/9618092

**Published:** 2015-12-06

**Authors:** Stefano Menini, Carla Iacobini, Claudia Blasetti Fantauzzi, Carlo M. Pesce, Giuseppe Pugliese

**Affiliations:** ^1^Department of Clinical and Molecular Medicine, “La Sapienza” University, Via di Grottarossa 1035-1039, 00189 Rome, Italy; ^2^DINOGMI, University of Genoa Medical School, 16132 Genoa, Italy

## Abstract

Galectin-3 is an important modulator of several biological functions. It has been implicated in numerous disease conditions, particularly in the long-term complications of diabetes because of its ability to bind the advanced glycation/lipoxidation end products that accumulate in target organs and exert their toxic effects by triggering proinflammatory and prooxidant pathways. Recent evidence suggests that galectin-3 may also participate in the development of obesity and type 2 diabetes. It has been shown that galectin-3 levels are higher in obese and diabetic individuals and parallel deterioration of glucose homeostasis. Two studies in galectin-3 knockout mice fed a high-fat diet (HFD) have shown increased adiposity and adipose tissue and systemic inflammation associated with altered glucose homeostasis, suggesting that galectin-3 negatively modulates the responsiveness of innate and adaptive immunity to overnutrition. However, these studies have also shown that impaired glucose homeostasis occurs in galectin-3 knockout animals independently of obesity. Moreover, another study reported decreased weight and fat mass in HFD-fed galectin-3 knockout mice. *In vitro*, galectin-3 was found to stimulate differentiation of preadipocytes into mature adipocytes. Altogether, these data indicate that galectin-3 deserves further attention in order to clarify its role as a potential player and therapeutic target in obesity and type 2 diabetes.

## 1. Introduction

Galectin-3, a chimera-type member of the galectin family, has been recently recognized as an important modulator of biological functions and an emerging player in the pathogenesis of common disease conditions, including cancer [1] and immune/inflammatory [2] and metabolic [3, 4] disorders. Considerable attention has been paid to the role of galectin-3 in the onset and progression of long-term complications of diabetes because of its ability to bind the advanced glycation end products (AGEs) and advanced lipoxidation end products (ALEs) that accumulate in target organs and exert their toxic effects by triggering proinflammatory and prooxidant pathways [5, 6]. With this respect, galectin-3 was shown to exert a protective effect toward the development of renal disease and atherosclerosis in experimental animal models [3, 4], that is, the opposite of the receptor for AGEs (RAGE), the first identified AGE receptor, which is known to mediate AGE/ALE-induced tissue injury [7] and to be upregulated in the absence of galectin-3 [3, 4]. Recently, galectin-3 has been implicated also in the development of metabolic disorders, such as obesity and type 2 diabetes, though data are still fragmentary [3]. This review article is concerned with current knowledge on the role of galectin-3 in metabolic disorders and the mechanism by which this lectin eventually modulates excess fat mass, adipose tissue and systemic inflammation, and the associated impairment in glucose regulation, with a view to highlighting the unresolved issues that deserve further research.

## 2. Galectin-3 Structure and Function

Galectin-3 is a 29- to 35-kDa protein consisting of two domains, the C-terminal carbohydrate recognition domain (CRD), with highly conserved residues between galectins, and the N-terminal domain, with a unique short end continuing into an intervening proline-glycine-alanine-tyrosine-rich repeat motif [8]. Galectin-3 is mainly located in the cytoplasm in quiescent cells, and in the nucleus in replicating cells [9]; it is also secreted into the extracellular space through a nonclassical secretory pathway [10]. Extracellular galectin-3 interacts with the *β*-galactoside residues of several glycoproteins via the CRD, and increasing concentrations of multivalent glycoprotein ligands drive formation of multimeric structures, which result in higher order lattices. The galectin-glycoprotein lattice has been shown to play a role in the regulation of receptor clustering, endocytosis, and signaling [11]. As a component of the cell surface lattice, galectin-3 also regulates the biogenesis of a subpopulation of clathrin-independent carriers (CLICs) involved in endocytosis of specific cargo proteins [12]. This function most likely represents the mechanism through which galectin-3 regulates tyrosine kinase and TGF-*β* receptors signaling, thereby controlling important cell functions such as cell migration, transdifferentiation, and fibrogenesis [11, 13]. Moreover, the galectin-3 lattice on the cell surface and its related function in CLIC biogenesis may play a role in AGE/ALE binding, internalization, and degradation [3]. Intracellular galectin-3 interacts with ligands mainly via peptide-peptide associations [8].

These structural features enable galectin-3 to participate in several cell functions via its binding to extracellular and intracellular proteins and make it a broad-spectrum biological response modifier [3, 5]. In addition to serving as an AGE/ALE receptor, galectin-3 is involved in pre-mRNA splicing [14], regulation of cell cycle [15] and Wnt/*β*-catenin signaling [16], and dual modulation of cell adhesion [17] and immune/inflammatory processes. In particular, galectin-3 has both pro- and anti-inflammatory effects, which depend on factors such as inflammatory setting and target cell/tissue [2, 3]. In acute inflammation, galectin-3 is a key component of the host defenses and promotes onset of the response against microbial infections and may affect immune/inflammatory cells by an autocrine/paracrine mechanism [2]. However, in chronic inflammation, a condition characterizing both diabetes and obesity, galectin-3 exerts some proresolution actions limiting further tissue injury and promoting repair. Relevant anti-inflammatory effects of galectin-3 include stimulation of T-cell apoptosis [18], inhibition of T-cell growth and T helper 1 differentiation [19], downregulation of T-cell receptor-mediated T-cell activation [20], induction of alternative macrophage activation [21], and promotion of efferocytosis of apoptotic neutrophils by macrophages [22]. Moreover, galectin-3 induces epithelial-mesenchymal transition and fibrogenesis, which may favor limitation of inflammation and wound healing [23], unless the fibrotic process proceeds beyond tissue repair [24, 25]. Finally, galectin-3 has been recently shown to restrain the inflammatory response that involves the Toll-like receptor- (TLR-) 2 and 4 and the proinflammatory cytokines interleukin- (IL-) 1-*β* and tumor necrosis factor-*α* in macrophages, an effect that contributes to delay the course of Wallerian degeneration in peripheral nerves [26], and to reduce the susceptibility to endotoxin shock [27]. Conversely, galectin-3/TLR-4 interaction has been reported to sustain microglia activation [28].

## 3. Galectin-3 in Obesity and Impaired Glucose Homeostasis

Obesity and type 2 diabetes are reaching epidemic proportions in the Western world [29] and call for novel pharmacological interventions to complement lifestyle modifications in preventing strategies. The parallel rise in the incidence and prevalence of these two conditions supports the concept that overweight and obesity are powerful risk factors for developing type 2 diabetes [30]. The mechanisms linking excess fat mass, especially at the visceral level, and impaired glucose regulation include adipose tissue macrophage infiltration and deranged lipid metabolism. These changes result in the release of cytokines and free fatty acids which cause insulin resistance and *β*-cell dysfunction [31]. Several proinflammatory and prooxidant signals have been implicated in the pathogenesis of obesity, including the AGES/ALEs through their receptor RAGE [32].

Recently, a wealth of human studies has provided evidence that galectin-3 levels are increased in subjects with obesity and type 2 diabetes, and animal studies have suggested that galectin-3 may be involved in the onset and progression of these metabolic disorders by acting primarily at the adipose tissue level.

### 3.1. Human Studies

In the general population, levels of circulating galectin-3 have been shown to correlate positively with age, prevalence of obesity, diabetes, hypercholesterolemia, and hypertension, markers of inflammation, and target organ damage [33, 34], thus indicating a strict relationship of galectin-3 with metabolic disorders and associated risk factors and complications.

**Figure 1:**
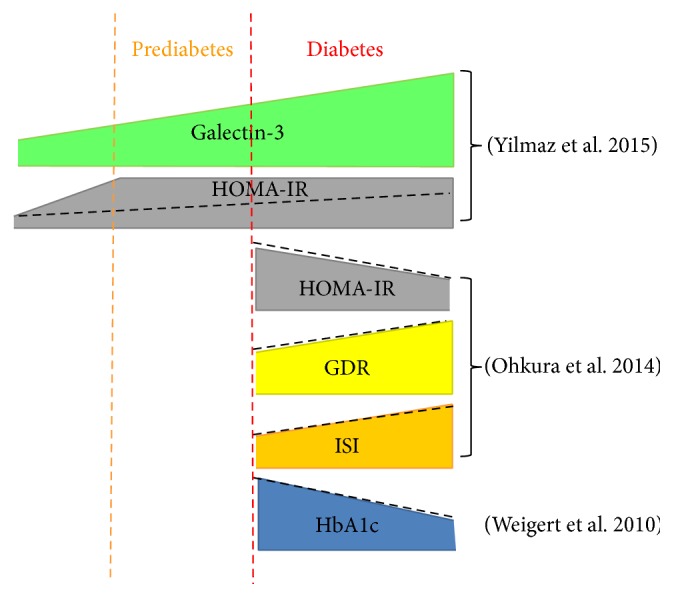
Summary of results of human studies on the role of galectin-3 in impaired glucose regulation and insulin sensitivity. Solid geometric figures indicate levels of each target, whereas dotted black lines indicate positive or negative correlation with galectin-3 levels. In the general population, galectin-3 levels are positively correlated with HOMA-IR, a measure of insulin resistance. Conversely, within the diabetic population galectin-3 levels are negatively correlated to HOMA-IR and HbA_1c_ levels and positively with GDR and ISI. HOMA-IR = homeostasis model assessment of insulin resistance; GDR = glucose disposal rate; ISI = insulin sensitivity index; HbA_1c_ = hemoglobin A_1c_.

A few studies have investigated the relationship between circulating levels of galectin-3, obesity and parameters of glucose metabolism, and insulin sensitivity in patients with diabetes (Figure 1). Weigert et al. [35] reported higher circulating galectin-3 levels in 30 overweight nondiabetic subjects and 30 patients with type 2 diabetes, in comparison with 23 normal-weight controls. All of them showed galectin-3 levels that correlated positively with body mass index (BMI), as well as, in a BMI-dependent manner, with leptin, resistin, IL-6, and age. In the diabetic population, circulating levels of galectin-3 correlated inversely with hemoglobin A_1c_ (HbA_1c_) and were higher in subjects with C-reactive protein (CRP) values above 5 mg/L. Finally, galectin-3 levels were higher in portal vein blood than in blood from hepatic and systemic veins. This observation suggests that the splanchnic region constitutes a major site for galectin-3 removal, a finding consistent with the scavenging role of this lectin in the hepatic clearance of circulating AGEs [36].

More recently, Yilmaz et al. [37] studied 174 subjects divided into three groups, nondiabetic, prediabetic, and diabetic, based on a 75-g oral glucose tolerance test. Galectin-3 levels were significantly higher in diabetic patients than in prediabetic and nondiabetic groups and higher in the prediabetic than in the nondiabetic group. Moreover, galectin-3 levels correlated with fasting plasma glucose (*r* = 0.787, *P* < 0.01), 2-hour plasma glucose (*r* = 0.833, *P* < 0.01), CRP (*r* = 0.501, *P* < 0.01), and homeostasis model assessment of insulin resistance (HOMA-IR) index (*r* = 0.518, *P* < 0.01). In multivariate logistic regression analysis galectin-3 was an independent predictor of diabetes. Moreover, in receiver operating characteristic analysis, a galectin-3 cut-off value of 803.55 pg/mL was found to diagnose diabetes with a sensitivity of 80.7% and a specificity of 85.5% (area under the curve = 0.912). Yilmaz et al. concluded that galectin-3 is a promising biomarker for early detection of prediabetes and diabetes onset and that it has a role in the progression from prediabetes to diabetes. However, data from this cross-sectional study cannot be construed as evidence of causality between galectin-3 changes and impaired glucose metabolism. In addition, calculation of HOMA-IR index is not the most appropriate method to estimate insulin resistance in this type of studies, where patients with poor glycemic control, low BMI, or severe *β*-cell dysfunction could be included [38].

A recent study from Ohkura et al. has investigated the relationship between galectin-3 and insulin sensitivity in 20 patients with type 2 diabetes through the euglycemic-hyperinsulinemic clamp, the gold standard for the assessment of insulin sensitivity, a meal tolerance test (MTT), and HOMA-IR index [39]. Galectin-3 levels correlated positively with the glucose disposal rate (*r* = 0.71, *P* < 0.001), insulin sensitivity index in MTT (*r* = 0.62, *P* < 0.005), and adiponectin concentration (*r* = 0.61, *P* < 0.05) and negatively with the HOMA-IR index (*r* = −0.52, *P* < 0.05) and fasting insulin concentration (*r* = −0.56, *P* < 0.01). In MTT, galectin-3 levels were not significantly associated with the areas under the curve of glucose, insulin, and the insulin/glucose ratio; a negative, but not significant, association was reported with insulin and the insulin/glucose ratio. These data, which should be taken with caution because of the small size of the study, prompted the authors to conclude that galectin-3 affects the concentration of insulin more than that of glucose and that increase of galectin-3 activity in diabetic subjects could improve insulin sensitivity.

These apparently contrasting results might be reconciled by claiming a role for galectin-3 upregulation as an adaptive mechanism to counteract the progression of metabolic derangement by favoring glucose disposal. In this view, galectin-3 levels would increase with development of obesity and diabetes, thus serving as a marker of these disorders, in which this lectin exerts a protective effect toward insulin resistance. This interpretation is consistent with the role of galectin-3 in favoring AGE/ALE disposal [3, 4], as a number of studies in nondiabetic individuals have shown that serum levels of AGEs or of their carbonyl precursors are independent correlates of insulin resistance, as assessed by HOMA-IR index [40].

### 3.2. Animal Studies

**Figure 2:**
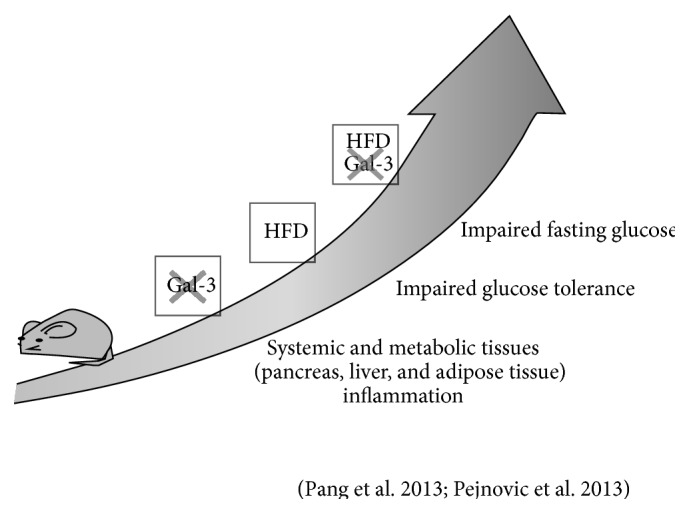
Summary of results of animal studies on the role of galectin-3 in deranged glucose homeostasis and “metabolic” inflammation. Like HFD, galectin-3 ablation induces glucose metabolism dysregulation and metainflammation. Galectin-3 ablation and HFD have cumulative effects in inducing metabolic and inflammatory alterations. Gal-3 = galectin-3; crossed Gal-3 = galectin-3 ablation; HFD = high-fat diet.

**Figure 3:**
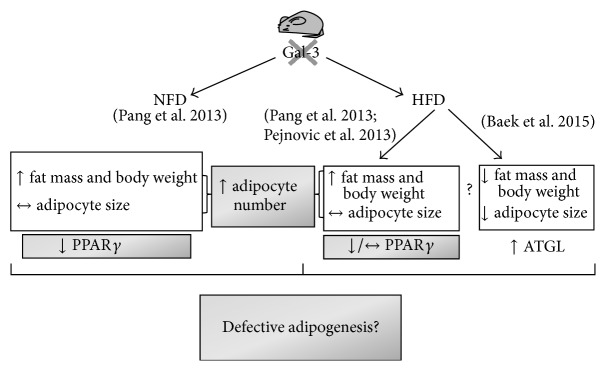
Summary of results of animal studies on the role of galectin-3 in obesity and adipogenesis. Structural and molecular features of adipose tissue of galectin-3 knockout mice, either fed with NFD or HFD. With NFD, galectin-3 ablation is associated with increased adiposity. However, fat cell size is not increased and PPAR*γ* expression is even reduced. With HFD, there are contrasting results on the effect of galectin-3 ablation on fat mass and body weight. However, galectin-3 ablation is always associated with defective size and/or impaired ability of adipocytes to respond with an enlargement to the fat overload. These structural features, together with defects in the expression of adipogenic and lipogenic markers (PPAR*γ* and ATGL), suggest a regulatory role for galectin-3 in adipogenesis. Gal-3 = galectin-3; crossed Gal-3 = galectin-3 ablation; NFD = normal-fat diet; HFD = high-fat diet; PPAR*γ* = peroxisome-proliferator-activated receptor *γ*; ATGL = adipose triacylglycerol lipase.

Several experimental studies have investigated the role of galectin-3 in the development of type 2 diabetes (Figure 2) and obesity (Figure 3).

Pejnovic et al. [41] and Pang et al. [42] reported increased fat accumulation and inflammation at the visceral adipose tissue and systemic level in association with altered glucose homeostasis in galectin-3 knockout mice fed a high-fat diet (HFD). Pejnovic et al. also found that galectin-3 knockout mice fed a HFD had increased inflammation in the pancreatic islets associated with accumulation of AGEs [41], which might be responsible for the impaired glucose regulation. This interpretation is consistent with a previous report showing that aminoguanidine prevents decreased glucose-stimulated insulin secretion in islets exposed to high glucose concentrations [43]. These data support the concept that galectin-3 primarily decreases the response of innate and adaptive immunity to overnutrition which leads to adipose tissue inflammation and oxidative stress, thus protecting against the obesity-associated type 2 diabetes [41, 42]. This view is consistent with a major anti-inflammatory effect of galectin-3 in chronic settings [3], the loss of which results in increased macrophage infiltration and release of proinflammatory and prooxidant cytokines at the adipose tissue level that enhances the metabolic derangements associated with fat accumulation. This interpretation is also in keeping with the finding that mice lacking RAGE exhibit an opposite response to a HFD, that is, reduced weight gain and inflammation [44]. However, these data do not explain the mechanism by which galectin-3 limits the expansion of adipose tissue and this interpretation has been challenged by other observations.

Firstly, both Pejnovic et al. [41] and Pang et al. [42] found that blood glucose levels were higher also in galectin-3 knockout fed a standard diet versus the corresponding wild type mice. Moreover, Pang et al. found that, in these animals, increased fat mass and systemic inflammation occurred after the development of altered glucose homeostasis, which was not accompanied by decreased insulin sensitivity [42], thus suggesting that galectin-3 modulates *β*-cell function, irrespective of obesity-related inflammation. However, there are conflicting data in the literature on this issue. In fact, previous studies showed that, in rat pancreatic islets exposed to IL-1*β*, galectin-3 is the most upregulated protein and that its overexpression protects the *β*-cells from the cytotoxic effect of IL-1*β* [45]. In contrast, galectin-3 appeared to be crucial for immune mediated *β*-cell damage, as galectin-3 knockout mice were resistant to multiple low doses of streptozotocin, a classical model of type 1 diabetes [46].

Secondly, at variance with these earlier studies, Baek et al. have recently reported that galectin-3 knockout mice had significantly lower body weight and epididymal white adipose tissue and higher expression of adipose triacylglycerol lipase, the rate-limiting enzyme in fat cell lipolysis, as compared with the corresponding wild type animals after 12-week feeding with a HFD [47]. Conversely, and again at variance with Pejnovic et al. [41] and Pang et al. [42], plasma glucose levels did not differ between the two genotypes, though glucose metabolism was not thoroughly investigated [47]. Interestingly, decreased body size and epididymal white adipose tissue were observed also in galectin-3 knockout mice fed a standard chow for 17 months, with no sign of inflammation [47]. The authors also showed that galectin-3 knockdown significantly reduced the differentiation of 3T3-L1 cells into mature fat cells and the expression of the master regulator of adipocyte differentiation peroxisome-proliferator-activated receptor *γ* (PPAR*γ*) as well as of CCAAT-enhancer-binding protein (C/EBP) *α* and C/EBP *β* [41]. Moreover, galectin-3 was found to interact with PPAR*γ* and to regulate its expression and transcriptional activation, thus suggesting that galectin-3 plays a direct role in adipogenesis through PPAR*γ* regulation [47]. Reduced lipid accumulation was paralleled by decreased fat cell enlargement, indicating that lower adiposity in galectin-3 knockout mice was not due to a decrease in fat cell number [47]. Indeed, Pang et al. found that the mRNA levels of PPAR*γ* were lower in galectin-3 knockout than in wild type mice and that the fat cell size did not differ between the two genotypes, irrespective of diet [42]. The results of the study of Baek et al., though preliminary, point to a stimulatory role of galectin-3 in adipogenesis, with the lack of this lectin resulting in reduced body weight gain in response to HFD and aging. In fact, in human and mouse adipose tissue, galectin-3 is expressed not only by macrophages but also by fat cells, where it is modulated during cell differentiation, with high level in the preadipocyte fraction and almost nil in differentiated fat cells [48]. Moreover, recombinant human galectin-3 was found to stimulate preadipocyte proliferation as well as DNA synthesis [48]. Unfortunately, Baek et al. did not investigate whether feeding a HFD induced adipose tissue and systemic inflammation [47], which appeared to parallel increased fat mass and to drive deranged glucose homeostasis in previous reports from Pejnovic et al. [41] and Pang et al. [42].

Altogether, these contrasting findings point to the need of further investigation on the effect of galectin-3 ablation on adipose tissue morphology and function and glucose homeostasis as well as on the response to a HFD.

## 4. Adipose Tissue in Type 2 Diabetes: Just a Matter of Quantity? The Role of Galectin-3

The studies in experimental animal models reviewed above have provided new insights in the relationships between adipocyte size and glucose homeostasis. Previous observations point to a critical role of lipid handling capacity of white adipose tissue in the development of type 2 diabetes. This is a key concept of the “adipose tissue expandability” theory, which suggests that increased size of adipocytes, close to a critical volume, favors diversion of lipids to other tissues, cell suffering, and consequent activation of proinflammatory and prooxidant pathways at the tissue and systemic level [49].

This theory has been also used to explain the paradox of the existence of a subset of individuals with normal insulin sensitivity despite being obese, on the one hand, and of lean insulin-resistant/diabetic subjects, on the other hand [50]. In fact, it has been proposed that white adipose tissue inflammation and associated insulin resistance are coupled to adipocyte hypertrophy regardless of fat mass and body weight. However, this traditional view has been recently challenged by the evidence that small, not enlarged, fat cells are associated with the onset of insulin resistance and inflammation in response to overfeeding [51]. Moreover, in obese subjects, small fat cells predominate in individuals with abnormal insulin resistance, whereas larger fat cells are more numerous in insulin-sensitive subjects [52]. Although no significant difference in the mean size of the larger cells was found between the insulin-sensitive and insulin-resistant individuals, insulin resistance was associated with a higher ratio of small to large cells [52]. A key point of this study is that the expression of genes related to fat cell differentiation and levels of adiponectin are significantly lower in the insulin-resistant group compared to the insulin-sensitive group, a finding suggesting that the increased number of small fat cells is the consequence of their inability of differentiating into a mature and functional phenotype [52]. Therefore, an alternative hypothesis is that the “metabolic buffer” function of the adipose tissue might be also disrupted by defective adipogenesis which, by reducing the plasticity of adipose tissue in terms of ability to store and metabolize lipids, decreases the threshold of critical volume at which adipocytes cannot properly cope with the metabolic demand.

The studies in galectin-3 knockout mice reviewed above showed that these animals (a) are more susceptible to type 2 diabetes when challenged with a HFD without developing adipocyte hypertrophy and (b) show spontaneous derangement of glucose homeostasis associated with smaller and less mature adipocytes. These data suggest that animals lacking galectin-3 respond to increased fat intake with adipocyte hyperplasia, as the hypertrophic response is impaired [42]. This interpretation is once again consistent with the opposite finding observed in RAGE null mice fed a HFD, which displayed smaller adipocytes than wild type littermates, but reduction in size was much less than reduction in body weight and fat mass, thus suggesting that the hyperplasic response was fundamentally impaired in these mice [44]. These observations make the galectin-3 knockout mice a suitable animal model to test the alternative hypothesis which attributes to small, immature adipocytes a major role in the development of obesity and associated derangement of glucose homeostasis.

## 5. Conclusions

Recently, galectin-3 has been implicated in the development of type 2 diabetes and obesity. Studies in humans have shown that galectin-3 concentration is higher in obese and diabetic individuals and that it increases in conjunction with unbridled glucose homeostasis. On the other hand, galectin-3 levels correlated positively with insulin sensitivity and negatively with HbA_1c_ levels in patients with type 2 diabetes. Taken together, these data suggest that galectin-3 is a surrogate marker, rather than a mediator of metabolic disorders, in which it might play a protective role, as part of an adaptive response. Moreover, galectin-3 appears to be a modulator of adipogenesis by stimulating differentiation of preadipocytes into mature adipocytes. The absence of fat cell enlargement in galectin-3 knockout mice suggests that this lectin might play a role in the storage capacity of the adipose tissue. Future studies should test the hypothesis that lack of galectin-3 accelerates disruption of glucose metabolism by affecting adipogenesis and lipid storage capacity.

In summary, modulation of galectin-3, an emerging all-out player in metabolic disorders, deserves further scientific attention as a novel marker and therapeutic avenue for the control of metabolic disorders such as diabetes and obesity.

## Abbreviations

AGEs: Advanced glycation end products
ALEs: Advanced lipoxidation end products
RAGE: Receptor for AGEs
CRD: Carbohydrate recognition domain
CLIC: Clathrin-independent carriers
IL: Interleukin
TLR: Toll-like receptor
BMI: Body mass index
HbA_1c_: Hemoglobin A_1c_
CRP: C-reactive protein
HOMA-IR: Homeostasis model assessment of insulin resistance
MTT: Meal tolerance test
HFD: High-fat diet
PPAR*γ*: Peroxisome-proliferator-activated receptor *γ*
C/EBP: CCAAT-enhancer-binding protein.

## References

[1] Newlaczyl A. U., Yu L.-G. (2011). Galectin-3—a jack-of-all-trades in cancer. *Cancer Letters*.

[2] Dumic J., Dabelic S., Flögel M. (2006). Galectin-3: an open-ended story. *Biochimica et Biophysica Acta—General Subjects*.

[3] Pugliese G., Iacobini C., Pesce C. M., Menini S. (2015). Galectin-3: an emerging all-out player in metabolic disorders and their complications. *Glycobiology*.

[4] Pugliese G., Iacobini C., Ricci C., Fantauzzi C. B., Menini S. (2014). Galectin-3 in diabetic patients. *Clinical Chemistry and Laboratory Medicine*.

[5] Iacobini C., Amadio L., Oddi G. (2003). Role of galectin-3 in diabetic nephropathy. *Journal of the American Society of Nephrology*.

[6] Pugliese G. (2008). Do advanced glycation end products contribute to the development of long-term diabetic complications?. *Nutrition, Metabolism and Cardiovascular Diseases*.

[7] Yan S. F., Ramasamy R., Schmidt A. M. (2008). Mechanisms of Disease: advanced glycation end-products and their receptor in inflammation and diabetes complications. *Nature Clinical Practice Endocrinology and Metabolism*.

[8] Barondes S. H., Cooper D. N. W., Gitt M. A., Leffler H. (1994). Galectins. Structure and function of a large family of animal lectins. *The Journal of Biological Chemistry*.

[9] Liu F.-T., Patterson R. J., Wang J. L. (2002). Intracellular functions of galectins. *Biochimica et Biophysica Acta—General Subjects*.

[10] Menon R. P., Hughes R. C. (1999). Determinants in the N-terminal domains of galectin-3 for secretion by a novel pathway circumventing the endoplasmic reticulum-Golgi complex. *European Journal of Biochemistry*.

[11] Dennis J. W., Nabi I. R., Demetriou M. (2009). Metabolism, cell surface organization, and disease. *Cell*.

[12] Lakshminarayan R., Wunder C., Becken U. (2014). Galectin-3 drives glycosphingolipid-dependent biogenesis of clathrin-independent carriers. *Nature cell Biology*.

[13] Partridge E. A., Le Roy C., Di Guglielmo G. M. (2004). Regulation of cytokine receptors by golgi N-glycan processing and endocytosis. *Science*.

[14] Dagher S. F., Wang J. L., Patterson R. J. (1995). Identification of galectin-3 as a factor in pre-mRNA splicing. *Proceedings of the National Academy of Sciences of the United States of America*.

[15] Yang R.-Y., Hsu D. K., Liu F.-T. (1996). Expression of galectin-3 modulates T-cell growth and apoptosis. *Proceedings of the National Academy of Sciences of the United States of America*.

[16] Shimura T., Takenaka Y., Fukumori T. (2005). Implication of galectin-3 in Wnt signaling. *Cancer Research*.

[17] Hughes R. C. (2001). Galectins as modulators of cell adhesion. *Biochimie*.

[18] Fukumori T., Takenaka Y., Yoshii T. (2003). CD29 and CD7 mediate galectin-3-induced type II T-cell apoptosis. *Cancer Research*.

[19] Morgan R., Gao G., Pawling J., Dennis J. W., Demetriou M., Li B. (2004). *N*-acetylglucosaminyltransferase V (Mgat5)-mediated *N*-glycosylation negatively regulates Th1 cytokine production by T cells. *Journal of Immunology*.

[20] Demetriou M., Granovsky M., Quaggin S., Dennis J. W. (2001). Negative regulation of T-cell activation and autoimmunity by Mgat5 N-glycosylation. *Nature*.

[21] MacKinnon A. C., Farnworth S. L., Hodkinson P. S. (2008). Regulation of alternative macrophage activation by galectin-3. *Journal of Immunology*.

[22] Karlsson A., Christenson K., Matlak M. (2009). Galectin-3 functions as an opsonin and enhances the macrophage clearance of apoptotic neutrophils. *Glycobiology*.

[23] González G. E., Cassaglia P., Noli Truant S. (2014). Galectin-3 is essential for early wound healing and ventricular remodeling after myocardial infarction in mice. *International Journal of Cardiology*.

[24] MacKinnon A. C., Gibbons M. A., Farnworth S. L. (2012). Regulation of transforming growth factor-*β*1-driven lung fibrosis by galectin-3. *American Journal of Respiratory and Critical Care Medicine*.

[25] Henderson N. C., Mackinnon A. C., Farnworth S. L. (2006). Galectin-3 regulates myofibroblast activation and hepatic fibrosis. *Proceedings of the National Academy of Sciences of the United States of America*.

[26] Mietto B. S., Jurgensen S., Alves L. (2013). Lack of galectin-3 speeds Wallerian degeneration by altering TLR and pro-inflammatory cytokine expressions in injured sciatic nerve. *European Journal of Neuroscience*.

[27] Li Y., Komai-Koma M., Gilchrist D. S. (2008). Galectin-3 is a negative regulator of lipopolysaccharide-mediated inflammation. *Journal of Immunology*.

[28] Burguillos M. A., Svensson M., Schulte T. (2015). Microglia-secreted galectin-3 acts as a Toll-like receptor 4 ligand and contributes to microglial activation. *Cell Reports*.

[29] Daar A. S., Singer P. A., Persad D. L. (2007). Grand challenges in chronic non-communicable diseases. *Nature*.

[30] Kodama S., Horikawa C., Fujihara K. (2014). Quantitative relationship between body weight gain in adulthood and incident type 2 diabetes: a meta-analysis. *Obesity Reviews*.

[31] Kahn S. E., Hull R. L., Utzschneider K. M. (2006). Mechanisms linking obesity to insulin resistance and type 2 diabetes. *Nature*.

[32] Schmidt A. M. (2015). The growing problem of obesity: mechanisms, consequences, and therapeutic approaches. *Arteriosclerosis, Thrombosis, and Vascular Biology*.

[33] de Boer R. A., van Veldhuisen D. J., Gansevoort R. T. (2012). The fibrosis marker galectin-3 and outcome in the general population. *Journal of Internal Medicine*.

[34] Ho J. E., Liu C., Lyass A. (2012). Galectin-3, a marker of cardiac fibrosis, predicts incident heart failure in the community. *Journal of the American College of Cardiology*.

[35] Weigert J., Neumeier M., Wanninger J. (2010). Serum galectin-3 is elevated in obesity and negatively correlates with glycosylated hemoglobin in type 2 diabetes. *Journal of Clinical Endocrinology and Metabolism*.

[36] Iacobini C., Menini S., Ricci C. (2011). Galectin-3 ablation protects mice from diet-induced NASH: a major scavenging role for galectin-3 in liver. *Journal of Hepatology*.

[37] Yilmaz H., Cakmak M., Inan O., Darcin T., Akcay A. (2015). Increased levels of galectin-3 were associated with prediabetes and diabetes: new risk factor?. *Journal of Endocrinological Investigation*.

[38] Kang E. S., Yun Y. S., Park S. W. (2005). Limitation of the validity of the homeostasis model assessment as an index of insulin resistance in Korea. *Metabolism*.

[39] Ohkura T., Fujioka Y., Nakanishi R. (2014). Low serum galectin-3 concentrations are associated with insulin resistance in patients with type 2 diabetes mellitus. *Diabetology and Metabolic Syndrome*.

[40] Song F., Schmidt A. M. (2012). Glycation and insulin resistance: novel mechanisms and unique targets?. *Arteriosclerosis, Thrombosis, and Vascular Biology*.

[41] Pejnovic N. N., Pantic J. M., Jovanovic I. P. (2013). Galectin-3 deficiency accelerates high-fat diet-induced obesity and amplifies inflammation in adipose tissue and pancreatic islets. *Diabetes*.

[42] Pang J., Rhodes D. H., Pini M. (2013). Increased adiposity, dysregulated glucose metabolism and systemic inflammation in galectin-3 KO mice. *PLoS ONE*.

[43] Kooptiwut S., Kebede M., Zraika S. (2005). High glucose-induced impairment in insulin secretion is associated with reduction in islet glucokinase in a mouse model of susceptibility to islet dysfunction. *Journal of Molecular Endocrinology*.

[44] Song F., del Pozo C. H., Rosario R. (2014). RAGE regulates the metabolic and inflammatory response to high-fat feeding in mice. *Diabetes*.

[45] Karlsen A. E., Størling Z. M., Sparre T. (2006). Immune-mediated *β*-cell destruction in vitro and in vivo—a pivotal role for galectin-3. *Biochemical and Biophysical Research Communications*.

[46] Mensah-Brown E. P. K., Al Rabesi Z., Shahin A. (2009). Targeted disruption of the galectin-3 gene results in decreased susceptibility to multiple low dose streptozotocin-induced diabetes in mice. *Clinical Immunology*.

[47] Baek J.-H., Kim S.-J., Kang H. G. (2015). Galectin-3 activates PPAR*γ* and supports white adipose tissue formation and high-fat diet-induced obesity. *Endocrinology*.

[48] Kiwaki K., Novak C. M., Hsu D. K., Liu F.-T., Levine J. A. (2007). Galectin-3 stimulates preadipocyte proliferation and is up-regulated in growing adipose tissue. *Obesity*.

[49] Virtue S., Vidal-Puig A. (2010). Adipose tissue expandability, lipotoxicity and the Metabolic Syndrome—an allostatic perspective. *Biochimica et Biophysica Acta—Molecular and Cell Biology of Lipids*.

[50] Badoud F., Perreault M., Zulyniak M. A., Mutch D. M. (2015). Molecular insights into the role of white adipose tissue in metabolically unhealthy normal weight and metabolically healthy obese individuals. *The FASEB Journal*.

[51] Johannsen D. L., Tchoukalova Y., Tam C. S. (2014). Effect of 8 weeks of overfeeding on ectopic fat deposition and insulin sensitivity: testing the ‘adipose tissue expandability’ hypothesis. *Diabetes Care*.

[52] McLaughlin T., Sherman A., Tsao P. (2007). Enhanced proportion of small adipose cells in insulin-resistant vs insulin-sensitive obese individuals implicates impaired adipogenesis. *Diabetologia*.

